# Diagnostic and prognostic value of urine biomarkers among women with dysfunctional voiding

**DOI:** 10.1038/s41598-022-10696-w

**Published:** 2022-04-22

**Authors:** Yuan-Hong Jiang, Jia-Fong Jhang, Han-Chen Ho, Yung-Hsiang Hsu, Hann-Chorng Kuo

**Affiliations:** 1 Department of Urology, Hualien Tzu Chi Hospital, Buddhist Tzu Chi Medical Foundation, Hualien, Taiwan; 2 Department of Urology, School of Medicine, Tzu Chi University, Hualien, Taiwan; 3 Department of Anatomy, Tzu Chi University, Hualien, Taiwan; 4 Department of Pathology, Hualien Tzu Chi Hospital, Buddhist Tzu Chi Medical Foundation and Tzu Chi University, Hualien, Taiwan

**Keywords:** Biomarkers, Urology

## Abstract

The current study aimed to investigate the diagnostic and prognostic value of urine biomarkers among female patients with dysfunctional voiding (DV). Urine samples were collected from 43 female patients with DV and 25 controls. Oxidative stress biomarkers (8-hydroxy-2-deoxyguanosine [8-OHdG], 8-isoprostane, and total antioxidant capacity [TAC]) and inflammatory markers (interleukin-1 beta [IL-1β], IL-2, IL-6, IL-8, tumor necrosis factor alpha, nerve growth factor, and brain-derived neurotrophic factor) levels were analyzed. In total, 26 patients with DV received further treatment with biofeedback pelvic floor muscle exercise or external urethral sphincter botulinum toxin A injections. Patients with DV had significantly higher urine 8-OHdG, IL-1β, IL-8, and brain-derived neurotrophic factor levels than controls. Both urine 8-OHdG and IL-1β levels were positively correlated with clinical symptoms. Patients with DV who had successful treatment outcomes had significantly lower pretreatment urine 8-isoprostane and TAC levels than those with unsuccessful outcomes. The pretreatment urine TAC level was the only independent predictor of successful treatment outcomes (odds ratio: 0.995). Compared with controls, female patients with DV had distinct urine oxidative stress biomarker and inflammatory marker profiles, which also mapped their clinical characteristics and treatment outcomes. These urine analytes might have diagnostic and prognostic values among female patients with DV.

## Introduction

During voluntary voiding, complete bladder emptying within a normal span of time is dependent on the initial relaxation of the external urethral sphincter (EUS) and subsequent sustained detrusor contraction. Abnormal activities of the EUS and/or pelvic floor muscle (PFM) during voiding can cause bladder outlet obstruction (BOO)^1^. Previously, in neurologically intact individuals, this condition is referred to as non-neurogenic bladder^2^. However, currently, it is more commonly called dysfunctional voiding (DV), and its occurrence among female individuals with voiding dysfunction has gained increasing attention recently^3–5^. The accurate diagnosis of DV relies on videourodynamic studies (VUDSs)^3^, which are invasive diagnostic procedures. The effective treatments for DV include biofeedback PFM exercise^6,7^ and EUS botulinum toxin A (BoNT-A) injections^8^. However, there is no consensus regarding the predictors of treatment outcomes.

In a retrospective analysis, 325 (17%) of 1914 women with refractory lower urinary tract symptoms who underwent VUDS were diagnosed with DV^4^. This condition is an important etiology of BOO among women^3^. In BOO progression, excessive oxidative stress and hypoxia-related inflammation resulting from cyclic ischemia–reperfusion injury involve in the bladder remodeling process^9^. The importance of oxidative stress markers (such as 8-hydroxy-2-deoxyguanosine [8-OHdG] and F2-isoprostane) and antioxidant biomarkers (such as total antioxidant capacity [TAC]) in BOO was assessed in animal model and human studies^10^. Urine is a highly attractive biospecimen for the biomarker analysis of different pathological diseases due to its non-invasive character^11^. Accordingly, urine oxidative stress and antioxidant biomarkers and their related inflammatory substances can be potential biomarkers of DV.

Despite some limitations, numerous research articles have reported about the association between urine biomarkers and lower urinary tract symptoms^12^. However, there is no study about female patients with DV. Therefore, the current research aimed to investigate the diagnostic and prognostic values of urine oxidative stress biomarkers and inflammatory markers levels among female patients with DV.

## Materials and methods

### Patients and investigation of the clinical characteristics

**Figure 1:**
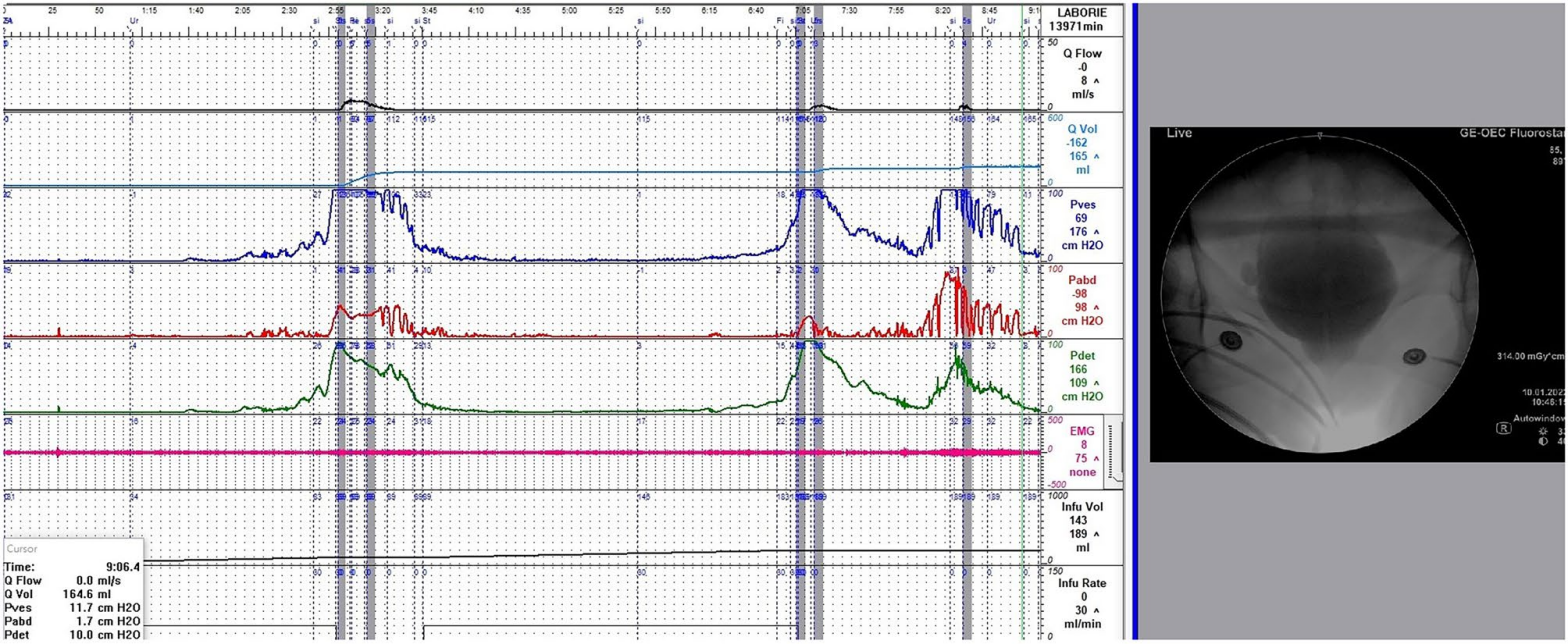
Videourodynamic study of dysfunctional voiding in a woman with an open bladder neck but a narrow membranous urethra (a spinning top appearance) on real-time fluoroscopy, increased urethral sphincter electromyography activities, a high detrusor pressure, and a low maximum flow rate during voiding.

From February 2015 to February 2021, we prospectively enrolled 46 female patients diagnosed with videourodynamic-confirmed DV at the department of urology of a single medical center. All patients underwent VUDS with the indication of refractory lower urinary tract symptoms. The diagnostic details were interpreted according to the International Continence Society terminology^1^. The following VUDS parameters were recorded: first sensation of bladder filling (FSF), bladder compliance, cystometric bladder capacity (CBC), detrusor voiding pressure (Pdet), maximal urinary flow rate (Qmax), corrected maximal urinary flow rate (cQmax, defined as Qmax/√CBC), voided volume (Vol), post-void residual volume (PVR), voiding efficacy (VE, defined as vol / CBC), and female bladder outlet obstruction index (BOOIf, defined as Pdet—2.2*Qmax)^13^. Patients with an open bladder neck but a narrow membranous urethra or PFM (a spinning top appearance) on real-time fluoroscopy, increased EUS electromyography activities, and low Qmax (with or without a high Pdet) during voiding were diagnosed with DV (Fig. 1). In addition, only patients without a history of neurological disease were eligible for this study. The exclusion criteria included genital prolapse, active urinary tract infection, interstitial cystitis, and previous surgery for urinary incontinence. In total, 25 women with stress urinary incontinence but without other significant storage or voiding dysfunction on VUDS were included as controls.

All patients were assessed with the International Prostate Symptom Score (IPSS), which included total IPSS, IPSS storage subscore (IPSS-S), and IPSS voiding subscore (IPSS-V). Biofeedback PFM exercise or EUS BoNT-A 100 U injection was recommended to patients with medically refractory DV according to the reported treatment protocols^7,8^. The treatment outcome was evaluated using the Global Response Assessment (GRA) as previous study^7^. Moreover, it was categorized as − 3, − 2, − 1, 0, 1, 2, and 3, which indicated markedly worse to markedly improved status) based on satisfaction 3 months after treatment. A successful outcome was defined as a GRA score of ≥ 2 (moderately and markedly improved).

### Assessment of urine biomarker levels

Urine samples were collected from all patients with DV and controls. Urine was self-voided by patients who had a full bladder sensation. Then, urinalysis was performed simultaneously to confirm an infection-free status before urine samples were stored. In total, 50-mL urine samples were placed on ice immediately and transferred to the laboratory for preparation. The samples were centrifuged at 1800 rpm for 10 min at 4 °C. The supernatant was separated into aliquots in 1.5-mL tubes (1 mL per tube) and was preserved in a freezer at − 80 °C. Before further analyses were performed, the frozen urine samples were centrifuged at 12,000 rpm for 15 min at 4 °C, and the supernatants were used for subsequent evaluations.

#### Quantification of 8-OHdG

The quantification of 8-OHdG in urine samples was performed in accordance with the manufacturer’s instructions (8-OHdG ELISA Kit, BioVision). Briefly, 50 μL of biotin-detection antibody working solution and 50 μL of sample were sequentially added to 96-well plates (panel kits), and the plates were incubated for 45 min at 37 °C. The well contents were removed, and the plates were washed three times with 350 μL of wash buffer. Next, 100 mL of horseradish peroxidase-streptavidin conjugate working solution was added to each well, and the plates were incubated for 30 min at 37 °C. The solution was discarded, and 350 μL of wash buffer was used to wash the plates five times. Next, 90 mL of TMB substrate was added into each well; incubation was then performed in the dark for 30 min at 37 °C. Finally, 50 μL of stop solution was added, and the plates were evaluated on the microplate reader at 450 nm. The median fluorescence intensities of the targets were analyzed to calculate the corresponding concentrations in the samples.

#### Quantification of 8-isoprostane

Eight-isoprostane belongs to F2-isoprostane class. The quantification of 8-isoprostane in urine samples was performed in accordance with the manufacturer’s instructions (8 isoprostane ELIZA kit, Enzo). Briefly, 50 μL of 8-iso-PGF2α conjugate solution, 50 μL of 8-iso-PGF2α antibody solution, and 100 μL of sample were sequentially added to 96-well plates (panel kits), and the plates were incubated 2 h at room temperature on a plate shaker at 500 rpm. The well contents were removed, and the plates were washed three times with 400 μL of wash buffer. In total, 200 mL of the pNpp substrate solution was added to each well, and the plates were incubated for 45 min at room temperature without shaking. Finally, 50 μL of stop solution was added, and the plates were read immediately at 405 nm. The median fluorescence intensities of the targets were analyzed to calculate the corresponding concentrations in the samples. The measurement of urine 8-isoprostane levels was standardized using urinary creatinine levels.

#### Quantification of TAC

The quantification of TAC in the samples was performed in accordance with the manufacturer’s instructions (Total Antioxidant Capacity Assay Kit, abcam). Briefly, 100 μL of Cu2 + working solution and 100 μL of sample were sequentially added to 96-well plates (panel kits), and the plates were incubated for 90 min at room temperature on a shaker protected from light. Finally, the plates were evaluated on the microplate reader at 570 nm. The median fluorescence intensities of the target were analyzed to calculate the corresponding TAC concentrations in the samples.

#### Evaluation of inflammatory substances

Inflammatory markers including cytokines and neurotrophins in the urine samples were assayed using commercially available microspheres with the Milliplex® human cytokine/chemokine magnetic bead-based panel kit (Millipore, Darmstadt, Germany).

Seven targeted analytes were used for the multiplex kit, and these included the following inflammatory cytokines: catalog number HCYTMAG-60 K (interleukin-1 beta [IL-1β], IL-2, IL-6, IL-8, and tumor necrosis factor alpha [TNFα]), catalog number HADK2MAG-61 K (nerve growth factor [NGF]), and catalog number HNDG3MAG-36 K (brain-derived neurotrophic factor [BDNF]). The following laboratory procedures for the quantification of these targeted analytes were similar to those reported in our previous studies^14,15^.

This study was approved by the Institutional Review Board and Ethics Committee of Buddhist Tzu Chi General Hospital (no. IRB107-37-A and IRB: 109-095-B). All methods/study were performed in accordance with the relevant guidelines and regulations. All patients with DV and controls were provided with information about the risk, rationale, procedures, ethics, and costs of this study, and they provided informed consent.

### Statistical analysis

Continuous variables were presented as means ± standard deviations and categorical variables as numbers and percentages. Outliers were defined as values outside the range between the means ± three standard deviations for each biomarker in either the study or the control group, and these were excluded from further analysis. The clinical data and urine biomarker levels between the study and control groups and between the different treatment outcome groups were analyzed using analysis of variance. Post-hoc power calculation was performed in the biomarker with significant difference between the study and control groups. Linear regression analysis with Pearson correlation was performed to determine the association between clinical characteristics and urine biomarker levels. To predict successful treatment outcomes, univariate and multivariate logistic regression model analyses were conducted with the calculation of odds ratio (OR). All calculations were performed using SPSS Statistics software for Windows version 20.0 (IBM Corp., Armonk, NY, the USA). A p value of < 0.05 was considered statistically significant.

## Results

The mean age of female patients with DV (n = 43) was 54.0 ± 14.1 (range: 24–77) years, and that of controls (n = 25) was 60.5 ± 10.6 (range: 41–68) years (Table 1). Based on the VUDS, patients with DV had a significantly lower CBC and Qmax and higher Pdet, PVR, and BOOIf than controls.

**Table 1:** Clinical characteristics of patients with DV and controls.

	DV group (n = 43)	Control group (n = 25)	P value
Age	54.0 ± 14.1	60.5 ± 10.6	0.069
IPSS-V	9.1 ± 7.2	1.4 ± 1.6	< 0.001
IPSS-S	6.0 ± 4.0	2.5 ± 2.0	0.001
IPSS	15.1 ± 9.9	3.9 ± 2.2	< 0.001
VUDS			
FSF	127.1 ± 54.4	164.6 ± 65.3	0.012
Bladder compliance	77.6 ± 84.1	158.1 ± 93.7	< 0.001
CBC	290.3 ± 125.6	379.0 ± 179.0	0.004
Pdet	47.79 ± 42.74	14.62 ± 7.19	0.001
Qmax	10.66 ± 6.85	19.41 ± 8.64	< 0.001
cQmax	0.63 ± 0.37	0.91 ± 0.39	< 0.001
Vol	232.7 ± 114.2	406.3 ± 143.7	< 0.001
PVR	57.7 ± 66.2	5.7 ± 12.4	< 0.001
VE	0.79 ± 0.23	0.98 ± 0.05	< 0.001
BOOIf	24.33 ± 47.82	− 30.41 ± 18.16	< 0.001

*DV* Dysfunctional voiding, *IPSS* International prostate symptom score, *IPSS-S* International prostate symptom score storage subscore, *IPSS-V* International prostate symptom score voiding subscore, *VUDS* Videourodynamic study, *FSF* First sensation of bladder filling, *CBC* Cystometric bladder capacity, *Pdet* Detrusor voiding pressure, *Qmax* Maximal urinary flow rate, *cQmax* Corrected maximal urinary flow rate, *Vol* Voided volume, *PVR* Post-void residual volume, *VE* Voiding efficacy, *BOOIf* Female bladder outlet obstruction index.

Table 2 shows the urine biomarker levels of patients with DV and controls. For each targeted analyte, the numbers of outliers in patients with DV and controls ranged from 0 to 2 and from 0 to 1, respectively, and all were < 5%. Patients with DV had higher urine 8-OHdG, IL-1β, IL-8, and BDNF levels than controls. Post-hoc power analysis reported 94.9, 89.3, 40.4, and 35.6% power (with alpha value of 0.05) in the evaluation of 8-OHdG, IL-1β, IL-8, and BDNF levels, respectively.

**Table 2:** Urine biomarker levels of patients with DV and controls.

Urine biomarkers^@^	DV group (n = 43)	Control group (n = 25)	P value
8-OHdG	32.09 ± 19.72 (0)	17.01 ± 14.59 (0)	0.002
8-isoprostane	12.94 ± 14.88 (1)	15.32 ± 15.34 (1)	0.098
TAC	607.28 ± 418.15 (2)	1006.32 ± 984.39 (0)	0.144
IL-1β	1.19 ± 1.43 (1)	0.48 ± 0.20 (1)	< 0.001
IL-2*	0.26 ± 0.21 (0)	0.73 ± 0.18 (0)	< 0.001
IL-6	2.21 ± 5.28 (2)	0.74 ± 0.35 (1)	0.729
IL-8	31.83 ± 65.21 (1)	12.68 ± 25.3 (0)	0.020
TNFα*	1.23 ± 0.33 (2)	0.69 ± 0.19 (0)	< 0.001
NGF*	0.22 ± 0.05 (1)	0.25 ± 0.06 (0)	0.033
BDNF	0.64 ± 0.15 (0)	0.58 ± 0.15 (0)	0.030

*DV* Dysfunctional voiding, *8-OHdG* 8-hydroxy-2-deoxyguanosine, *TAC* Total antioxidant capacity, *TNFα* Tumor necrosis factor alpha, *NGF* Nerve growth factor, *BDNF* Brain-derived neurotrophic factor.

(): number of outliers.

*: Mean values of the study group that were below the minimum detectable concentrations as per the assay manufacturer.

@: units: all pg/mL, except for ng/mL in 8-OHdG and mmol/μL in TAC.

Table 3 depicts the correlation coefficients between urine biomarker levels and the clinical characteristics of patients with DV. Urine 8-OHdG and IL-1β levels were positively correlated with the clinical symptom scores, and urine 8-isoprostane and TAC levels were negatively associated with GRA in patients with DV. There were also significant correlations between VUDS parameters and urine biomarker levels (including TAC and BDNF).

**Table 3:** Correlation coefficient (r value) between urine biomarker levels and the clinical characteristics of patients with DV.

	Urine biomarkers*						
	8-OHdG	8-isoprostane	TAC	IL-1β	IL-6	IL-8	BDNF
**Clinical symptoms**							
IPSS-V	0.455	n.s	n.s	0.522	n.s	n.s	n.s
IPSS-S	0.289	n.s	n.s	n.s	n.s	n.s	n.s
IPSS	0.438	n.s	n.s	0.438	n.s	n.s	n.s
**VUDS**							
FSF	n.s	n.s	n.s	n.s	n.s	n.s	n.s
Bladder compliance	n.s	n.s	n.s	n.s	n.s	n.s	− 0.261
CBC	n.s	n.s	0.275	n.s	n.s	n.s	n.s
Pdet	n.s	n.s	n.s	n.s	n.s	n.s	n.s
Qmax	n.s	n.s	0.272	n.s	n.s	n.s	n.s
cQmax	n.s	n.s	n.s	n.s	n.s	n.s	n.s
Vol	n.s	n.s	0.298	n.s	n.s	n.s	n.s
PVR	n.s	n.s	n.s	n.s	n.s	n.s	n.s
VE	n.s	n.s	n.s	n.s	n.s	n.s	n.s
BOOIf	n.s	n.s	n.s	n.s	n.s	n.s	n.s
**Treatment outcomes (n = 26)**							
GRA	n.s	− 0.440	− 0.605	n.s	n.s	n.s	n.s

*DV* Dysfunctional voiding, *8-OHdG* 8-hydroxy-2-deoxyguanosine, *TAC* Total antioxidant capacity, *VEGF* Vascular endothelial growth factor, *NGF* Nerve growth factor, *BDNF* Brain-derived neurotrophic factor, *IPSS* International prostate symptom score, *IPSS-S* International prostate symptom Score storage subscore, *IPSS-V* International prostate symptom score voiding subscore, *VUDS* Videourodynamic study, *FSF* First sensation of bladder filling, *CBC* Cystometric bladder capacity, *Pdet* Detrusor voiding pressure, *Qmax* Maximal urinary flow rate, *cQmax* Corrected maximal urinary flow rate, *Vol* Voided volume, *PVR* Post-void residual volume, *VE* Voiding efficacy, *BOOIf* Female bladder outlet obstruction index, *GRA* Global response assessment; *n.s.* Not significant.

*: Mean analyte volumes of the study group that were below the minimum detectable concentrations as per the assay manufacturer were excluded from further analysis.

**Figure 2:**
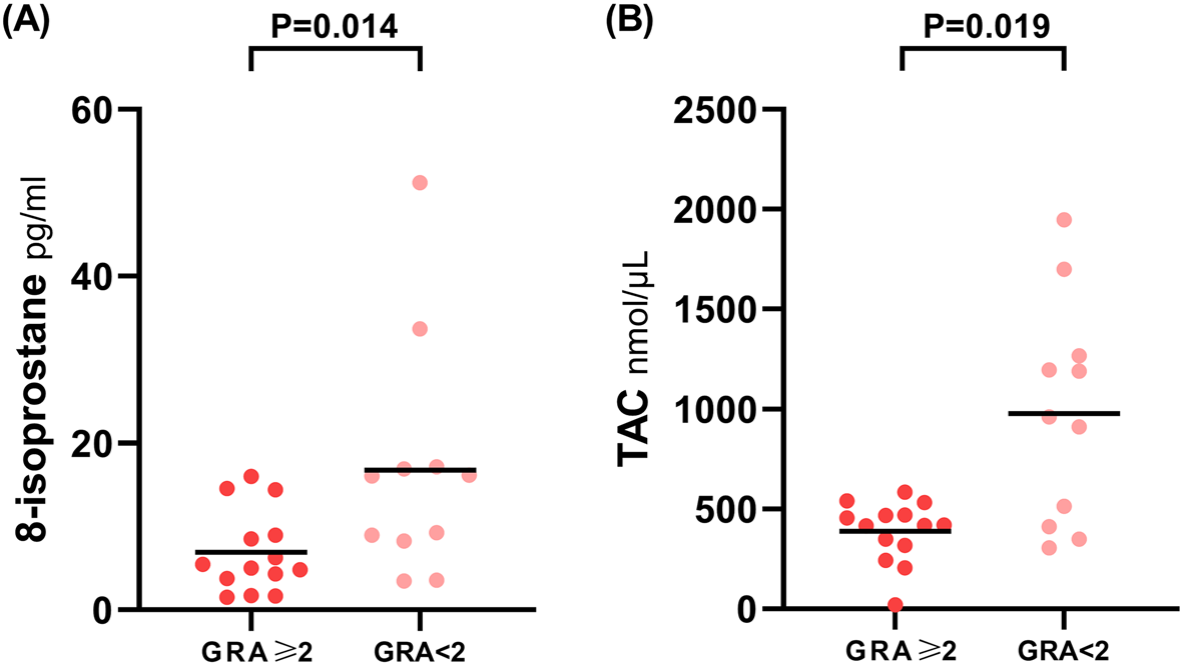
Difference in urine biomarker levels in patients with dysfunctional voiding who presented with different treatment outcomes. Patients with dysfunctional voiding who presented with a GRA score of ≥ 2 had significantly lower pretreatment urine 8-isoprostane (6.94 ± 4.92 vs 16.80 ± 14.19 pg/mL, *p*0.019) and TAC (388.31 ± 151.73 vs 977.63 ± 549.86 mmol/μL, *p* = 0.014) levels than those with a GRA score of < 2.

In total, 26 patients with DV received further treatment, with a total of 12 biofeedback PFM exercises and 14 EUS BoNT-A injections. Three months after treatment, 15 (57.7%) patients with DV had a successful outcome (as evidenced by a GRA score of ≥ 2). Table 4 shows the pretreatment clinical characteristics and urine biomarker levels of patients with DV with different treatment outcomes (GRA score of ≥ and < 2), and there were no significant differences in terms of pretreatment clinical symptoms or VUDS parameters. However, patients with DV who presented with a GRA score of ≥ 2 had significantly lower pretreatment urine 8-isoprostane (6.94 ± 4.92 vs 16.80 ± 14.19 pg/mL, p = 0.019) and TAC (388.31 ± 151.73 vs 977.63 ± 549.86 mmol/μL, p = 0.014) levels than those with a GRA score of < 2 (Table 4, and Fig. 2). In addition, the biofeedback PFM exercise group had a higher rate of successful outcome than the EUS BoNT-A injection group.

**Table 4:** Pretreatment clinical characteristics and urine biomarker levels of patients with DV who presented with different treatment outcomes.

	GRA score of ≥ 2 (n = 15)	GRA score of < 2 (n = 11)	P value
Age	48.4 ± 13.66	55.82 ± 12.38	0.212
IPSS-V	9.67 ± 7.10	11 ± 7.11	0.567
IPSS-S	6 ± 3.82	6 ± 4.43	0.875
IPSS	15.67 ± 10.2	17 ± 10.09	0.658
**VUDS**			
FSF	124.7 ± 60.2	128.0 ± 40.8	0.603
Bladder compliance	52.4 ± 77.9	79.7 ± 68.5	0.102
CBC	256.2 ± 110.0	319.6 ± 121.2	0.126
Pdet	60.07 ± 63.73	50.55 ± 37.18	0.662
Qmax	8.00 ± 4.05	11.50 ± 10.24	0.481
cQmax	0.49 ± 0.22	0.65 ± 0.54	0.755
Vol	202.2 ± 95.5	239.8 ± 120.4	0.551
PVR	54.0 ± 42.4	79.8 ± 71.2	0.343
VE	0.76 ± 0.26	0.73 ± 0.25	0.621
BOOIf	42.47 ± 64.65	25.25 ± 49.58	0.500
**Urine biomarkers**^**@**^			
8-OHdG	31.94 ± 19.39 (0)	42.01 ± 19.35 (0)	0.154
8-isoprostane	6.94 ± 4.92 (1)	16.80 ± 14.19 (0)	0.019
TAC	388.31 ± 151.73 (1)	977.63 ± 549.86 (0)	0.014
IL-1β	0.86 ± 0.13 (1)	0.85 ± 0.25 (0)	0.365
IL-2*	0.15 ± 0.03 (1)	0.20 ± 0.09 (0)	0.111
IL-6	4.06 ± 13.12 (1)	3.93 ± 8.40 (0)	0.511
IL-8	17.78 ± 22.3 5(0)	12.77 ± 16.05 (1)	0.677
TNFα*	1.24 ± 0.19 (1)	1.31 ± 0.15 (0)	0.443
NGF*	0.20 ± 0.03 (0)	0.22 ± 0.06 (1)	0.502
BDNF	0.64 ± 0.10 (0)	0.62 ± 0.08 (0)	0.751
**Treatment**			0.014
Biofeedback PFM exercise	10	2	
EUS BoNT-A injections	5	9	

*DV* Dysfunctional voiding, *GRA* Global response assessment, *IPSS* International prostate symptom score, *IPSS-S* International prostate symptom Score storage subscore, *IPSS-V* International prostate symptom score voiding subscore, *VUDS* Videourodynamic study, *FSF* First sensation of bladder filling, *CBC* Cystometric bladder capacity, *Pdet* Detrusor voiding pressure, *Qmax* Maximal urinary flow rate, *cQmax* Corrected maximal urinary flow rate, *Vol* Voided volume, *PVR* Post-void residual volume, *VE* Voiding efficacy, *BOOIf* Female bladder outlet obstruction index, *8-OHdG* 8-hydroxy-2-deoxyguanosine, *TAC* Total antioxidant capacity, *TNF* Alpha, tumor necrosis factor α, *NGF* Nerve growth factor, *BDNF* Brain-derived neurotrophic factor, *PFM* Pelvic floor muscle, *EUS* External urethral sphincter, *BoNT-A* Botulinum toxin A.

(): number of outliers.

*: Mean values of the study group that were below the minimum detectable concentrations as per the assay manufacturer.

@: units: all pg/mL, except for ng/mL in 8-OHdG and mmol/μL in TAC.

Univariate logistic regression analysis revealed that the pretreatment urine TAC levels (OR: 0.994) and the treatment strategies with biofeedback PFM exercise (OR: 9.000) were predictors of successful treatment outcomes (Table 5). Multivariate logistic regression analysis revealed that the pretreatment urine TAC level (OR: 0.995) was the only independent predictor of successful treatment.

**Table 5:** Univariate and multivariate logistic regression analyses for predicting successful treatment outcomes in patients with DV.

	GRA score of ≥ 2					
	Univariate analysis			Multivariate analysis		
	OR	95% CI	P value	OR	95% CI	P value
**Urine biomarkers**						
8-isoprostane	0.853	0.726–1.003	0.054			
TAC	0.994	0.989–1.000	0.033	0.995	0.990–1.000	0.049
**Treatment**						
Biofeedback PFM exercise (ref. EUS BoNT-A injection)	9.000	1.386–58.443	0.021	7.742	0.691–86.731	0.097

*DV* Dysfunctional voiding, *GRA* Global response assessment, *TAC* Total antioxidant capacity, *PFM* Pelvic floor muscle, *EUS* External urethral sphincter, *BoNT-A* Botulinum toxin A.

## Discussion

To the best of our knowledge, this study first investigated the value of urine oxidative stress biomarker and inflammatory marker levels among female patients with DV. These patients had distinct urine oxidative stress biomarker and inflammatory marker profiles compared with controls. Moreover, there were significant correlations between urine biomarker levels and clinical characteristics in this patient group, and the pretreatment urine TAC level was an independent factor for predicting successful treatment outcomes. Urine component analysis, which is a noninvasive approach, might provide important clinical information in the establishment of diagnosis, mapping of clinical characteristics, and prediction of prognosis in female patients with DV. These urine analytes might have diagnostic and prognostic values and could be biomarkers in female patients with DV.

One recent review summarized studies about the biomarkers of oxidative stress, which included 8-OHdG, F2-isoprostane, and TAC, in BOO. Results revealed the significant association between oxidative stress and BOO-related urinary dysfunctions^10^. Moreover, 8-OHdG is a stable end product of DNA oxidation ^16^. F2-isoprostane is a chemically stable compound and is formed by free radical-induced perioxidation of arachidonic acid^17^. TAC reflects the cumulative effect of all antioxidants from different endogenous antioxidative defense systems against harmful activities caused by oxidative stress^18^. Tissue hypoxia and hypoxia-induced inflammatory pathways play critical roles in disease progression and bladder remodeling in BOO ^9,19^. These oxidative stress biomarkers might be applied in DV, which is an important etiology of BOO.

In redox signaling and oxidative stress, reactive oxygen species affect the release of proinflammatory cytokines including IL-1β, TNFα, and interferon beta, thereby resulting in related immune responses and metabolism^20^. In a previous rabbit study, the 8-OHdG levels in the urine, plasma, and tissue increased, and the plasma TAC levels decreased under partial BOO^21^. In a rat study of chronic bladder ischemia induced by atherosclerosis, the study group had significantly higher urine 8-OHdG and proinflammatory cytokine (TNFα, IL-6, and IL-8) levels in the bladder tissue than controls^22^. In this study, patients with DV had significantly higher urine 8-OHdG, IL-1β, and IL-8 levels than controls. In addition, urine 8-OHdG and IL-1β levels were positively correlated with the symptom scores in patients with DV. Elevated urine 8-OHdG and IL-1β levels might reflect more oxidative stress and increasing hypoxia-related inflammation in the urinary bladder among patients with DV, which corresponds to clinical symptom severity. The noninvasive investigation of urine specimen might provide information about clinical symptoms and the pathology in the urinary bladder. Both urine 8-OHdG and IL-1β levels might have diagnostic values among female patients with DV. Our results were in accordance with the findings about the importance of elevated oxidative stress biomarker and proinflammatory cytokine levels in chronic bladder ischemia ^22^.

F2-isoprostatne is a reliable indicator of oxidative stress and can be detected in all tissues and fluids^10,16^. In a mouse bladder model of partial BOO, the assessment of F2-isoprostane in the bladder tissue reflects the progression of oxidative stress^19^. However, studies about the presence of isoprostane in the urine in lower urinary tract diseases are limited. Eight-isoprostane belongs to F2-isoprostane class. In this study, the urine 8-isoprostane levels did not significantly differ between patients with DV and controls. However, patients with DV who presented with successful treatment outcomes had significantly lower pretreatment urine 8-isoprostane levels, and the pretreatment urine 8-isoprostane level was found to be negatively correlated with GRA. Patients with DV who presented with lower urine 8-isoprostane levels might achieve better therapeutic effects. Although both 8-OHdG and 8-isoprostane are oxidative stress biomarkers, their respective levels in the urine might reflect unique underlying metabolic processes and clinical values. In patients with DV, urine 8-OHdG and 8-isoprostane levels could possibly have diagnostic and prognostic values, respectively.

TAC is an available biomarker for assessing the antioxidant potential of body fluids including urine^23,24^. Urine TAC levels might be affected by infection, renal status, systemic diseases, nutrition and supplemental intervention, and lifestyle factors^24^. The interplay between oxidative stress and antioxidative stress is dynamic and complex^20^. Therefore, the association between the expressions of oxidative stress biomarker (e.g., 8-OHdG and 8-isoprostane) and TAC in the urine cannot be definitely reversed, as shown in the current study. Different stages of pathological conditions can possibly have distinct oxidative stress and antioxidative stress profiles. In this study, the urine TAC levels of patients with DV was non-significantly lower than that of controls, and a low urine TAC level is an independent predictor of successful outcome, thereby indicating its prognostic value. Currently, the treatment outcomes of patients with DV varied without convincing predictors^7,8^, and the influencing factors might be complex and multifactorial. The urine oxidative stress and antioxidative stress biomarker profiles might be the summarized index reflecting the overall current status of lower urinary tract functions in individuals.

The current study had several limitations. First, it included women. Hence, sex bias might have existed. Second, although the design of the current study was similar to that of previous ones^14,15^, all controls presented with SUI. Third, even though oxidative stress biomarkers are more stable compounds than inflammatory cytokines, both might have intra-individual variations. Fourth, the overall hypoxia and oxidative stress status of the bladder was not completely attributed to DV. Both systemic inflammatory diseases and comorbidities and local bladder insults might affect the expressions of these urine substances. Finally, we excluded extreme values from analysis, as in previous studies^14,15^. However, the percentage of outliers was low.

## Conclusion

Compared with controls, female patients with DV had distinct urine oxidative stress biomarker and inflammatory marker profiles. They had elevated urine 8-OHdG and IL-1β levels, which were also positively correlated with clinical symptoms. The pretreatment urine TAC level was an independent predictor of successful treatment outcomes. These urine analytes might have diagnostic and prognostic values and could be used as biomarkers of DV among women.

## Data Availability

The datasets used and/or analyzed during the current study available from the corresponding author on reasonable request.
